# Experimental and Numerical Analysis of a Pd–Ag Membrane Unit for Hydrogen Isotope Recovery in a Solid Blanket

**DOI:** 10.3390/membranes13060578

**Published:** 2023-06-01

**Authors:** Vincenzo Narcisi, Luca Tamborrini, Luca Farina, Gessica Cortese, Francesco Romanelli, Alessia Santucci

**Affiliations:** 1 Fusion and Technology for Nuclear Safety and Security Department, ENEA, Via E. Fermi 45, 00044 Frascati, Italy; vincenzo.narcisi@enea.it (V.N.); luca.farina@enea.it (L.F.); gessica.cortese@enea.it (G.C.); 2 Department of Industrial Engineering, University of Rome “Tor Vergata”, Via del Politecnico 1, 00133 Rome, Italy

**Keywords:** fusion, DEMO, Helium-Cooled Pebble Bed, fuel cycle, Tritium Extraction and Recovery System, permeator, permeability, permeation, HyFraMe, code validation

## Abstract

The interest of the fusion community in Pd–Ag membranes has grown in the last decades due to the high value of hydrogen permeability and the possibility of continuous operation, making it a promising technology when a gaseous stream of hydrogen isotopes must be recovered and separated from other impurities. This is the case of the Tritium Conditioning System (TCS) of the European fusion power plant demonstrator, called DEMO. This paper presents an experimental and numerical activity aimed at (i) assessing the Pd–Ag permeator performance under TCS-relevant conditions, (ii) validating a numerical tool for scale-up purposes, and (iii) carrying out a preliminary design of a TCS based on Pd–Ag membranes. Experiments were performed by feeding the membrane with a He–H_2_ gas mixture in a specific feed flow rate ranging from 85.4 to 427.2 mol h^−1^ m^−2^. A satisfactory agreement between experiments and simulations was obtained over a wide range of compositions, showing a root mean squared relative error of 2.3%. The experiments also recognized the Pd–Ag permeator as a promising technology for the DEMO TCS under the identified conditions. The scale-up procedure ended with a preliminary sizing of the system, relying on multi-tube permeators with an overall number ranging between 150 and 80 membranes in lengths of 500 and 1000 mm each.

## 1. Introduction

In the mid-term, nuclear fusion represents one of the possible answers to the energy and environmental crises. The reaction releases a large amount of energy without emissions of greenhouse gases. Options to make fusion are several and span different reactions and confining principles. Among all the options, the one based on the deuterium (D) and tritium (T) reaction confined via strong magnetic fields is the main reference for the activities carried out in the EUROfusion program [1]. In this context, one of the main needs is represented by the availability of the required fuel, that is the D–T mixture. While deuterium can be extracted from seawater, tritium is very rare, its stock is mainly linked to the operation of CANDU reactors [2], and the available supply is currently estimated at twenty kilos [3]. For such reasons, a fundamental aspect of the fusion research program is the development of the so-called breeding blanket (BB) whose function is to produce the tritium consumed in the D–T reaction plus an additional amount necessary for the start-up of future machines. Inside the BB, tritium production occurs from the interaction between the neutrons escaping the plasma and the lithium contained in the blanket material.

**Figure 1:**
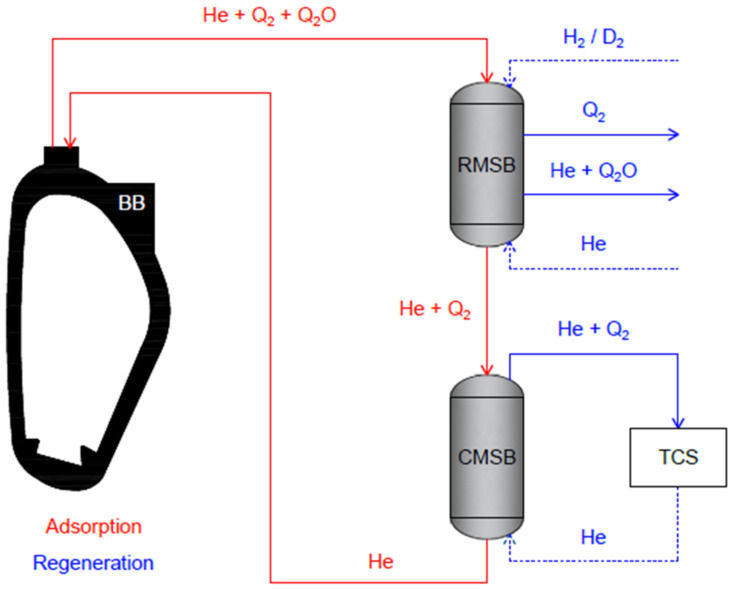
Schematic view of the TERS operating principle and TCS functionality.

The Helium-Cooled Pebble Bed (HCPB) represents one of the promising tritium BB concepts for the demonstration fusion power plant named DEMO [4,5]. Such a blanket uses ceramic pebbles made of lithium orthosilicate (Li_4_SiO_4_) or lithium metatitanate (Li_2_TiO_3_) as tritium breeder, pebbles of beryllium as neutron multiplier, and helium as primary coolant [6,7]. The tritium produced in the pebbles is released by purging with a helium stream doped with protium, from which it has to be opportunely recovered and purified in a closed loop, namely the Tritium Extraction and Recovery System (TERS). Currently, the reference process for the TERS relies on the sequential use of Reactive Molecular Sieve Beds (RMSB) and Cryogenic Molecular Sieve Beds (CMSB) [8]. A schematic view of the TERS operating principle is given in the diagram of Figure 1. Two operating phases can be distinguished: adsorption and regeneration. During the adsorption phase, the purge gas made of helium and hydrogen isotopes (Q = H, D, and T) in molecular (Q_2_) and water (Q_2_O) forms enters the RMSB for Q_2_O trapping, and then the CMSB for Q_2_ trapping. In the regeneration phase, Q_2_O and Q_2_ are released from the RMSB and the CMSB, respectively, thanks to appropriate regeneration procedures. The major contribution in terms of Q_2_ throughput derives from the regeneration of CMSB, where the trapped Q_2_ containing a helium amount of 5–10 mol% is released. This helium amount must be removed before sending the Q_2_ stream to the Isotope Separation System (ISS) and such function is in charge of the Tritium Conditioning System (TCS).

This work presents the results of an experimental and numerical activity carried out to assess the efficiency and the preliminary design of a Pd-based membrane unit for the TCS, required to separate the helium from the stream leaving the CMSB during its regeneration.

Membrane gas separation presents specific advantages compared to conventional separation processes, such as low environmental impact, low energy consumption, and simple operation. The selection of the material plays a key role in the separation efficiency. For instance, due to its high helium permeability, Hyflon AD60X is used for the helium production process [9]. Instead, the use of Pd-based technologies is quite consolidated in the fusion community [10] and in the Molten Salt Reactor (MSR) belonging to the Generation IV nuclear power plants [11]. Palladium is characterized by high values of hydrogen permeability and its alloy with silver improves its mechanical properties [10,12], so Pd–Ag membranes are a reliable technology for the separation of impurities from a gaseous stream of hydrogen isotopes. In fact, they represent a reference for the processing of the tokamak exhaust gases [13]. More generally, the use of Pd–Ag membranes in the fuel cycle of a fusion device is largely desired since these technologies operate continuously, thus reducing the inventory of tritium, the radioactive isotope of hydrogen.

The novelty of this work is that it investigates the use of Pd–Ag membranes inside the TCS belonging to the DEMO fuel cycle. The activity starts with an experimental campaign for assessing the membrane performance in hydrogen separation under operating conditions relevant for the TCS. Then, the experimental results are used to validate a numerical tool previously developed. Finally, a preliminary dimensioning of the membrane unit required for the HCPB TCS, obtained with the validated tool, is presented.

## 2. The Tritium Conditioning System

The fuel cycle of a fusion power plant is responsible for refueling the plasma, and for reprocessing and storing the stream coming from the plasma chamber along with all the tritiated effluents present in the plant. During the pre-concept design phase, Day et al. [14] developed a novel three-loop-based architecture for the DEMO fuel cycle aiming at minimizing tritium inventory in the system, promoting a continuous operation, and avoiding hold-ups of tritium in its processes.

The innovative architecture consists of the Direct Internal Recycling Loop (DIRL), acting as a bypass of the tritium plant systems for a large fraction of the plasma exhaust stream, the Inner Tritium Plant Loop (INTL), reprocessing all gases coming from the plasma chamber that are not recycled through the DIRL, and the Outer Tritium Plant Loop (OUTL), responsible for processing all tritiated streams not included in the circulated fuel.

In this paper, more emphasis is given to the OUTL, called to reprocess multiple streams in different tritium concentrations and chemical forms. Among these, the one coming from the BB represents the major tritium contribution to the OUTL. Referring to the solid blanket concept, the higher throughput expected from the TERS is that coming from the regeneration of CMSB, estimated by Cristescu and Draghia as 10 Nm^3^ h^−1^ consisting in 5–10 mol% of He and the rest Q_2_ [8]. The TCS has the central function of preparing this effluent for the isotope separation, and removing helium from the Q_2_ stream. Keeping in mind the main driver for the development of the novel fuel cycle architecture (i.e., tritium inventory minimization) and considering the major tritium contribution to the OUTL, the TCS should rely on continuous operation with the aim of limiting tritium immobilization. For this reason, and exploiting the high values of hydrogen permeability, a Pd–Ag permeator is selected as the most suitable technology.

## 3. The Experimental Campaign

### 3.1. Experimental Apparatus

**Figure 2:**
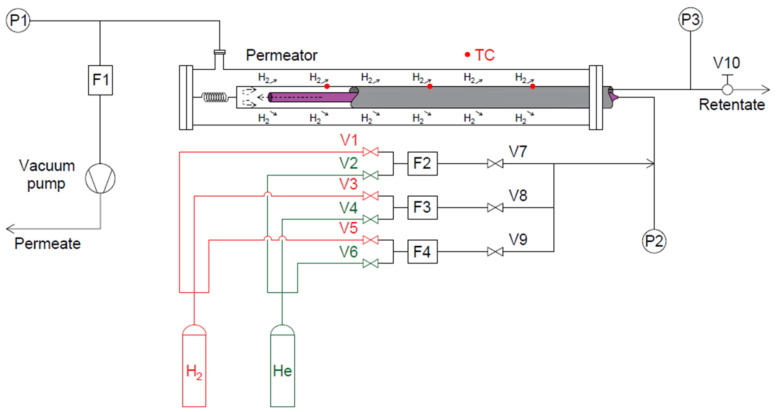
Schematic view of the HyFraMe test facility: main components and instrumentation.

The capabilities of Pd–Ag membranes were investigated in the HyFraMe (Hydrogen Frascati Membrane) experimental facility, designed and operated at the ENEA Frascati research center [15]. Figure 2 presents the schematic view of the test section, showing the main components along with the instrumentation for control and monitoring.

The permeator is the leading component of the apparatus and is composed of three concentric tubes. The inner stainless steel tube (purple in Figure 2, refer to the online version of the paper for colored figures) constitutes the permeator’s feed line, through which a proper gas mixture is injected into the unit. Once it reaches the outlet edge, the mixture flows through the annulus between the inner and the intermediate tubes (grey in Figure 2), namely the lumen side of the permeator. The intermediate tube constitutes the membrane made of Pd–Ag: its length is equal to 500 mm while the diameter and thickness are equal to 10 mm and 113 µm [15], respectively. Flowing along the lumen side, hydrogen is free to permeate through the membrane whereas the rest of the mixture, namely the retentate, exits the unit and is released through the V10 needle valve. The permeated flow, called permeate, is collected in the annulus between the membrane and the outer tube (i.e., the shell of the unit), namely the shell side of the permeator, and is vented through the vacuum pump.

The permeability *Pe* (mol m^−1^ s^−1^ Pa^−0.5^) of a dense membrane is described by Sieverts’ law:

(1) $$Pe=\frac{J\cdot th}{\Pi }$$

where *J* is the permeated flux (mol m^−1^ s^−1^), *th* is the membrane wall thickness (m), and *Π* (Pa^0.5^) represents the driving force of the permeation phenomenon, expressed as:

(2) $$\Pi =\sqrt{p_{H2,\;lumen}}-\sqrt{p_{H2,\;shell}}$$

where *p_H_*_2, *lumen*_ and *p_H_*_2, *shell*_ are the lumen-side and shell-side hydrogen partial pressures (Pa), respectively. Furthermore, the dependence of *Pe* on the temperature can be expressed by an Arrhenius-type behavior:

(3) $$Pe=Pe_{0}\cdot e^{-\frac{E_{a}}{R\cdot T}}$$

where *Pe*_0_ is the pre-exponential factor (mol m^−1^ s^−1^ Pa^−0.5^), *E_a_* is the apparent activation energy (J mol^−1^), *R* is the gas constant (8.314 J mol^−1^ K^−1^), and T is the temperature (K).

Given the above-mentioned features, in the HyFraMe set-up, the driving force of the permeation phenomenon is provided by the vacuum pump, connected to the shell side, and by the V10 needle valve, controlling the lumen-side pressure. Furthermore, a proper operative temperature is ensured by the membrane’s direct resistive heating, as presented by Tosti et al. [16]. Membrane temperature is monitored with three type K thermocouples (TC, red circles in Figure 2) installed on the membrane itself. The controlling and monitoring system is completed by the F1 mass flow meter, measuring the permeate flow rate, the F2, F3, and F4 mass flow controllers, providing the feed mixture to the permeator, the P1 pressure transducer for the shell-side vacuum measurement, and the P2 and P3 absolute pressure transducers for inlet and outlet lumen-side pressure acquisition, respectively. The specifications of the instrumentation are summarized in Table 1.

The figure of merit adopted in this work for the assessment of the permeator performance is the permeation efficiency *η*, evaluated under specific operative conditions with the following formula:

(4) $$\eta =\frac{\Gamma_{H2,\;perm}}{\Gamma_{H2,\;feed}}\cdot 100$$

where *Γ_H_*_2*,feed*_ and *Γ_H_*_2*,perm*_ are the feed and permeated hydrogen flow rates, respectively. In the following, the permeation efficiency is correlated to the main operative parameters, namely the feed flow rate, the membrane temperature, and the lumen-side pressure.

### 3.2. Experimental Test Matrix

The experimental campaign was carried out to (i) collect experimental data in a wide range of flow rates and compositions necessary for the validation of a numerical tool and (ii) to experimentally investigate the performance of a Pd–Ag permeator unit under relevant conditions for the TCS. For these purposes, two experimental test matrices were identified and are presented in Table 2 and Table 3, where the selected operative conditions are reported for the V-series (i.e., code validation purpose) and for the TCS-series (i.e., TCS relevance) experiments, respectively.

Twenty-five tests, each one repeated twice to assess repeatability, were carried out in the V series, imposing a membrane temperature of 623 K and lumen-side pressure of 200 kPa. Shell-side pressure is not directly controlled but depends on the pumping rate of the vacuum system. An average value of 30 kPa was observed throughout the whole V-series test matrix. The specific feed flow rate reported in Table 2 is the mixture feed flow per unit of permeation surface. The compositions selected for the experiments are ensured by operating the parallel mass flow controller connected to proper gas tanks (see Figure 1).

In the TCS series, different lumen-side pressures and membrane temperatures are investigated, aiming at deriving optimal operative conditions for the TCS. For this purpose, in comparison with the V series, the analysis was more interested in the low specific feed flow rates. The mixture composition derives from the TERS CMSB regeneration, in accordance with Cristescu and Draghia [8]. An overall number of 80 tests were performed, each one repeated twice for the repeatability assessment.

## 4. Results

In 2019, Antunes et al., presented a numerical tool conceived for the scaling-up studies of Pd–Ag permeators. The model relies on the finite element method and was developed to calculate the permeation efficiency of palladium membranes depending on geometry and operative conditions [17]. The numerical tool includes both the bulk and the surface contributions to permeation, although the latter can be neglected for thick membranes (i.e., *th* > 3 µm) and/or high hydrogen partial pressure [16]. Both conditions are verified in the present experimental campaign, thus surface contribution is neglected in the simulation.

The numerical tool will help the dimensioning of permeators for several sub-systems of the DEMO fuel cycle asked to work under a wide range of operative conditions [13]. For this purpose, the V-series tests aim at expanding the validation process of the model performed in 2019 [17] to a wider range of flow rates and compositions. The many input parameters required for the analysis are geometric characteristics of the membrane (i.e., length, diameter, and thickness), feed flow rates, membrane temperature, and operative pressures. Furthermore, the *Pe_0_* and *E_a_* quantities must be provided as input parameters. For this purpose, the values 2.45 × 10^−8^ mol m^−1^ s^−1^ Pa^−0.5^ and 3332 J mol^−1^ were measured in a permeability test carried out in HyFraMe and used for the analysis. Such values are in agreement with data found in the literature [17].

**Figure 3:**
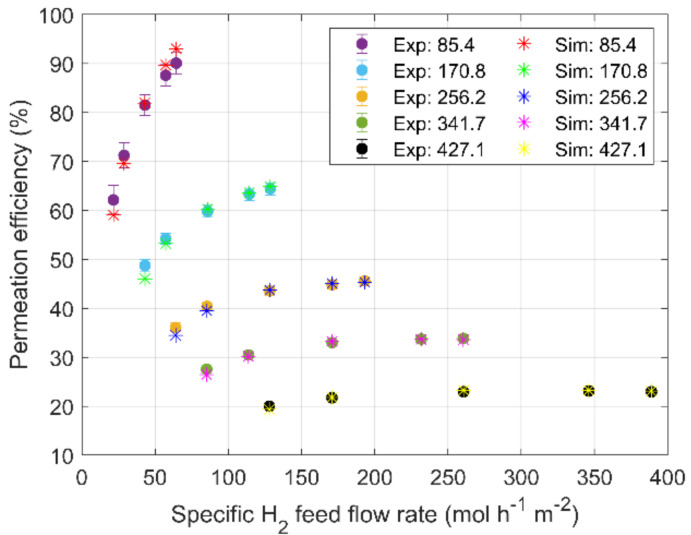
Comparison between permeation efficiency experimentally evaluated (circles) and calculated with numerical tool (asterisks) vs. specific feed flow rate of H_2_. Five sets of data for different specific mixture feed flow rates are expressed in mol h^−1^ m^−2^ in the legend.

The main outcomes of the validation analysis are presented in Figure 3, where the experimentally observed permeation efficiencies (circles) are compared with simulation results (asterisks) for different H_2_-specific feed flow rates (*x-axis*), that is the H_2_ feed flow rate per unit of permeation area (mol h^−1^ m^−2^). All the experiments exhibited good repeatability, giving a negligible standard deviation in comparison with the accuracy of the equipment. For this reason, the experimental data are treated according to the uncertainties derived from the equipment’s technical handbook, then following the propagation analysis.

As shown in Figure 3, the numerical tool provides a satisfactory qualitative prediction of the permeation efficiency in the whole V-series test matrix. Permeation efficiency increases when lowering the mixture feed flow rates, exhibiting the highest values for the case of 85.4 mol h^−1^ m^−2^. In this condition, the spatial velocity is the lowest, ensuring the longest residence time for the hydrogen into the unit. For a fixed value of the feed mixture flow rate (in Figure 3, circles with the same color), it is observed that the permeation efficiency increases with the increase in H_2_-specific flow, i.e., with the increase in lumen-side hydrogen partial pressure. As a matter of fact, a higher lumen-side hydrogen partial pressure leads to an improved permeation driving force *Π* (see Equation (2) for the definition). This is true for the cases from 85.4 to 256.2 mol h^−1^ m^−2^, whereas a plateau at approximately 35% and 25% is observed for the cases of 341.7 and 427.1 mol h^−1^ m^−2^, respectively. A threshold of 150 mol h^−1^ m^−2^ can be individuated for the H_2_-specific flow rate, over which the permeated flow rate increases almost linearly with the H_2_ feed flow rate, thus the permeation efficiency remains almost constant. As reported by Huang et al. [11], a phenomenon known as concentration polarization occurs when inert gas concentration, such as He in the present case, is high enough to form a boundary layer over the permeation surface that reduces hydrogen permeation. In the present analysis, such a phenomenon does not seem so relevant since the numerical tool provides satisfactory results without implementing any formulation of the concentration polarization. The same consideration can be shown for superficial effects which, given the experimental conditions (i.e., high hydrogen partial pressure) and membrane thickness, can be neglected.

Furthermore, when fixing the H_2_-specific flow rate, Figure 3 shows the decrease in permeation efficiency by increasing the feed mixture flow rate. As a matter of fact, keeping the same lumen-side absolute pressure, the increase in the mixture feed flow rate leads to the decrease in the lumen-side hydrogen partial pressure and, thus, in the permeation driving force.

All the observed phenomena are well reproduced with the numerical tool, and the discrepancy with experimental data is comparable with the measurement uncertainty. The reliability of the simulations has been quantitatively assessed with the Root Mean Squared Relative Error (*RMSRE*), calculated as:

(5) $$RMSRE\;\left(\%\right)=100\cdot \sqrt{\frac{1}{N}\overset{N}{\underset{i=1}{\sum }}{\left(\frac{\eta_{sim,i}-\eta_{exp,i}}{\eta_{exp,i}}\right)}^{2}}$$

where *N* is the total number of observations, and subscripts *sim* and *exp* stand for simulation results and experimental acquisitions, respectively. The analysis has shown a satisfactory *RMSRE* equal to 2.3% over the whole V-series test matrix.

Along with code validation, V-series tests provide useful outcomes for the definition of the TCS-series test matrix. Figure 3 shows low permeation efficiency when the HyFraMe permeator is operated with high specific feed flow rates. Thus, in such conditions, the permeator would not respect the target efficiency suitable for the TCS. Therefore, the TCS series has been focused on specific feed flow rates lower than 170.8 mol h^−1^ m^−2^.

**Figure 4:**
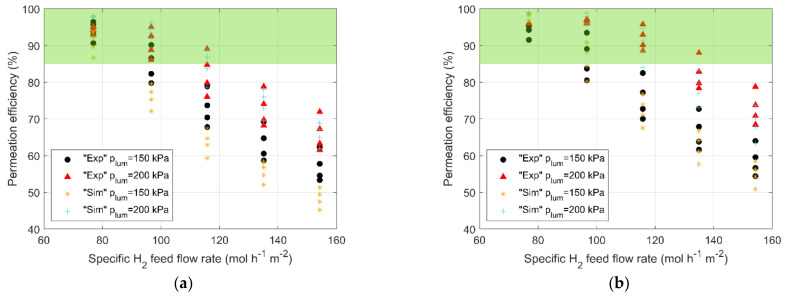
Comparison between permeation efficiency obtained in TCS-series experiments and simulation results for two mixture compositions relevant for DEMO HCPB BB TCS: (**a**) 90% H_2_ and 10% He; (**b**) 95% H_2_ and 5% He. Sensitivity analysis conducted over lumen-side pressure (150 and 200 kPa) and membrane temperature (see Table 3) to achieve permeation efficiency higher than 85%.

Figure 4 summarizes the main outcomes of the TCS-series experiments, comparing them with simulation results. Experimental error bars are not reported since they are below 2% for all the cases and are not visible in the scale of the plots. The permeation efficiency, evaluated with Equation (3), is plotted vs. the specific hydrogen feed flow rate for two lumen-side pressures (*p_lum_*) and four membrane temperatures. It is important to remember that higher membrane temperature leads to higher permeability and, thus, higher permeation efficiency. Regarding the shell-side pressure, an average value of 35 kPa was measured for all the tests belonging to the TCS series. Figure 4a,b reports the case of 90% H_2_ and 10% He, and 95% H_2_ and 5% He, respectively.

It is worth emphasizing the role of the TCS and the relevance of the permeation efficiency: TCS receives streams coming from the TERS CMSB regeneration and, after separating hydrogen isotopes from the He carrier gas, it returns helium with residual hydrogens, depending on permeation efficiency, to the TERS for further regeneration of CMSB. The higher the content of hydrogen isotopes in the returning stream is, the lower the regeneration efficiency of that bed, leading to higher tritium inventory and more regeneration phases. So, it would be convenient to maximize the TCS permeation efficiency, taking into account the size and the energy requirement of the TCS itself.

A target efficiency higher than 85% can be considered reasonable in this preliminary phase of the design. The green box in Figure 4 highlights the multiple combinations of operative parameters that ensure this target. Looking at the experimental outcomes, considering an inlet composition of 90% H_2_ and 10% He, the maximum allowable specific feed flow rate of the mixture is 128.1 mol h^−1^ m^−2^ (note that Figure 4 reports only the H_2_-specific feed flow rate on the *x-axis*), even though high lumen-side pressure (200 kPa) and high membrane temperature (673 K) are needed. Because of the presence of tritium in the TCS inlet stream (about 1% of mole fraction [8]), for safety concerns, it could be convenient to operate with reduced pressure. Furthermore, it is worth noticing that direct resistive heating should be avoided for a multi-tube module managing tritium. In this case, an external shell-side heating system should be foreseen, along with a pre-heating of the feed stream if needed, leading to high energy consumption and more complexity of the whole system in case of pre-heating. A lower temperature should be preferred, reducing the maximum allowable specific flow rate of the feed mixture below 106.8 mol h^−1^ m^−2^ (corresponding to the specific H_2_ feed flow rate of 96.12 mol h^−1^ m^−2^) for the case with lumen-side pressure of 200 kPa and inlet composition of 90% H_2_ and 10% He. Assuming a lumen-side pressure of 150 kPa, the same feed flow rate requires a membrane temperature of 623 K to ensure the target efficiency of 85%. Lowering the feed flow rate to 85.4 mol h^−1^ m^−2^ allows operation at low temperatures. The same qualitative outcomes were experienced for the case of 95% H_2_ and 5% He. In this case, the higher permeation efficiency, compared with the previous case, fulfills a higher specific feed flow rate (see Figure 4b).

The experimental outcomes of the TCS series have highlighted the possibility of using a Pd–Ag permeator to fulfill TCS scopes and have determined the operative conditions to ensure a permeation efficiency higher than 85%. Moreover, the same experimental data were used for further validation of the numerical tool, under TCS-relevant conditions. All the TCS-series operating conditions were simulated, obtaining a satisfactory agreement between experimental data and numerical outcomes (see Figure 4). Major discrepancies are highlighted for the higher flow rates with a lumen-side pressure of 150 kPa, where the numerical tool underestimates the permeation efficiency by approximately 10% (see Figure 4a). For the rest of the cases, the discrepancy is kept below 5%. The quantitative analysis, summarized in Table 4 in terms of *RMSRE*, confirms the qualitative assessment. It is worth emphasizing that for the cases suitable for the TCS (i.e., permeation efficiency higher than 85%), a satisfactory agreement is obtained and, thus, the numerical tool can be considered reliable for a TCS scale-up procedure.

## 5. Design and Optimization of the DEMO HCPB BB TCS

This section presents the preliminary dimensioning and the design optimization of the TCS relying on Pd–Ag membranes. The experimental outcomes constitute the starting point for the scale-up, and the validated numerical tool is used for the optimization of geometrical and operational parameters.

**Figure 5:**
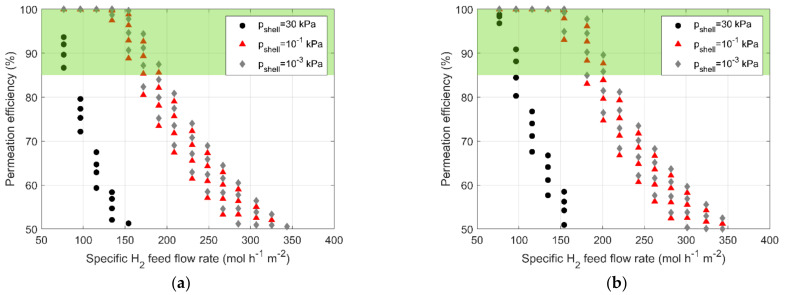
Permeation efficiency vs. specific H_2_ feed flow rate for three shell-side pressures (30 kPa circles, 10^−1^ kPa triangles, and 10^−3^ diamonds) and four different temperatures between 523 and 673 K: (**a**) 90% H_2_ and 10% He; (**b**) 95% H_2_ and 5% He.

The experimental campaign has investigated the effect of a specific feed flow rate and lumen-side pressure on the permeation efficiency, keeping the shell-side pressure at around 30 kPa. The pumping system installed in the facility does not allow higher vacuum levels, thus, relying upon the validated numerical tool, the first objective of the design process is to assess the possibility of using a low- and medium-vacuum condition (10^−1^ and 10^−3^ kPa, respectively) to improve permeator performance. The results of such analysis are presented in Figure 5, where the permeation efficiency calculated with the numerical tool is reported vs. the specific hydrogen feed flow rate. All the simulations are carried out assuming three different shell-side pressures (i.e., 30 kPa, corresponding to the HyFraMe vacuum level, 10^−1^ kPa, corresponding to the low vacuum, and 10^−3^ kPa, corresponding to the medium vacuum) and each one is repeated with the membrane temperature ranging between 523 and 673 K. The case with lumen-side pressure of 150 kPa is considered, for the compositions of 90% H_2_ and 10% He (Figure 5a) and 95% H_2_ and 5% He (Figure 5b). The analysis is also carried out for the case with 200 kPa, providing the same qualitative outcomes. For the sake of briefness, the latter is not reported in the present paper.

A significant improvement in permeator performance is observed by reducing shell-side pressure from 30 to 10^−1^ kPa, for both compositions. It allows the possibility of increasing the specific feed flow rate while keeping the target permeation efficiency of 85%. For both compositions, the specific hydrogen feed flow rate can be increased up to 211 mol h^−1^ m^−2^, accepting a membrane temperature of 673 K.

On the other hand, a shell-side pressure of 10^−3^ kPa does not lead to remarkable improvements and does not justify the eventual addition of complexity and the energy-demanding increase.

As a result of the computational activity and of the experimental campaign, the main operative parameters to be adopted in the TCS have been derived and summarized in Table 5. The values selected for the membrane temperature and the lumen-side pressure derive from a compromise between the size, the energy demand, and the safety of the TCS. Regarding the shell-side pressure, the value has been optimized with a numerical tool whereas the design-specific hydrogen flow rate has been chosen to respect the target permeation efficiency (>85%) for both compositions assumed for the TCS feed.

Once the operative conditions are defined, the scale-up procedure consists in evaluating the number of membranes required to process the streams coming from the TERS CMSB regeneration. The tubes are supposed to be arranged in a multi-tube module composed of 10 membranes, based on the design presented by Incelli et al. [18]. Thus, the total number of tubes is calculated assuming the design-specific feed flow rate reported in Table 5 and considering a total flow rate of 10 Nm^3^ h^−1^ that must be processed by the TCS [8]. The obtained number is rounded up to a multiple of 10 and the needed number of multi-tube modules is derived. Tube diameter and thickness are assumed equal to 10 mm and 110 μm, respectively, whereas three membrane lengths are considered: 500 mm, 750 mm, and 1000 mm. A further increase in the length is not recommended since the permeation phenomenon leads to elongation of the Pd–Ag tube proportional to its length, which could cause stress on the surface and risk membrane rupture. The size and the characteristics of the required modules are summarized in Table 6 and are valid for both compositions considered in the analysis.

## 6. Conclusions and Future Perspectives

An experimental and numerical investigation of Pd–Ag membranes has been presented in the paper, focusing on relevant conditions for the DEMO TCS. The experimental campaign, carried out in the HyFraMe test facility, has provided data for code validation and for the assessment of the permeator’s performance.

The validation procedure has consisted in the comparison between experimental data and simulation outcomes, showing a satisfactory qualitative agreement with an RMSRE of 2.3% for the V series, and between 4.04% and 11.47% for the TCS series.

Starting from the experimental outcomes of the TCS series, the validated numerical tool is firstly used to optimize the operative conditions required to fulfill a target permeation efficiency of 85%, and then, to scale up the membrane unit for TCS requirements. It consists of multi-tube modules (from 8 to 15 depending on the tubes’ length) connected in parallel. Each multi-tube module is composed of ten membranes installed in parallel. The present scale-up method refers to the experimental outcomes obtained using a single-tube permeator. Nevertheless, the validity of the scale-up procedure must be confirmed, and this will be the objective of future experimental campaigns that will be carried out with the multi-tube mock-up designed and constructed at the ENEA Frascati research center.

## Figures and Tables

**Table 1:** Instrumentation: model, measurement range, and accuracy.

Item	Model	Range	Accuracy
F1, F2, F3, F4	MKS, GE50A series	Depending on test	±1% of reading between 20% and 100% of full scale; ±0.2% of full scale between 2% and 20% of full scale
P1	MKS, 910 DualTrans™	1 × 10^−5^–1500 Torr	±0.75% of reading in the operative range
P2, P3	MKS, Baratron^®^ Type 722B	10^5^–10^6^ Pa	±0.5% of reading
TC1, TC2, TC3	Type-K	Up to 1073 K	±1.5 K

**Table 2:** V-series experimental test matrix: feed flow rate and mixture composition.

Test ID	Specific Feed Flow Rate (mol h^−1^ m^−2^)	Composition (%)	
		H_2_	He
From V-1 to V-25	85.4, 170.8, 256.2, 341.7, 427.1	25.00	75.00
		33.33	66.67
		50.00	50.00
		66.67	33.33
		75.00	25.00

**Table 3:** TCS-series experimental test matrix: feed flow rate and mixture composition, lumen-side pressure, and membrane temperature.

Test ID	Composition (%)		Lumen-Side Pressure (kPa)	Temperature (K)	Specific Feed Flow Rate (mol h^−1^ m^−2^)
	H_2_	He			
From TCS-1 to TCS-40	90	10	150, 200	523, 573, 623, 673	85.4
					106.8
					128.1
					149.5
					170.8
From TCS-41 to TCS-80	95	5	150, 200	523, 573, 623, 673	85.4
					106.8
					128.1
					149.5
					170.8

**Table 4:** Quantitative analysis for the validation procedure of the TCS-series experiments: *RMSRE* between experimental data and simulation outcomes.

Composition	Lumen-Side Pressure (kPa)	RMSRE (%)
90% H_2_ and 10% He	150	11.47
	200	4.97
95% H_2_ and 5% He	150	3.46
	200	4.04

**Table 5:** Operative conditions of the DEMO HCPB BB TCS relying on a Pd–Ag membrane.

Parameter	Composition	
	90% H_2_–10% He	95% H_2_–5% He
Membrane temperature (K)	573	573
Lumen-side pressure (kPa)	150	150
Shell-side pressure (kPa)	10^−1^	10^−1^
Design-specific H_2_ flow rate (mol h^−1^ m^−2^)	172	181.5
Permeation efficiency	85.32%	88.29%

**Table 6:** Preliminary sizing of the DEMO HCPB BB TCS.

Parameter	Tube Length (mm)		
	500	750	1000
Total number of tubes	150	100	80
Number of tubes per module	10	10	10
Shell diameter	DN 90	DN 90	DN 90
Total number of modules	15	10	8

## Data Availability

The data presented in this study are available on request from the corresponding author. The data are not publicly available due to restrictions from the funding institution.
